# Adjunctive steroid in HIV-negative patients with severe *Pneumocystis* pneumonia

**DOI:** 10.1186/1465-9921-14-87

**Published:** 2013-08-28

**Authors:** Virginie Lemiale, Alexandre Debrumetz, Alexandra Delannoy, Corinne Alberti, Elie Azoulay

**Affiliations:** 1 Medical Intensive Care Unit, Saint Louis Teaching Hospital, 1 Avenue Claude Vellefaux, Paris 75010, France; 2 AP-HP, hopital Robert Debré, Unité d'epidémiologie clinique, F-75010 Paris, France; 3 Université Paris 7, PRES Sorbonne Paris Cité, F-75010 Paris, France

**Keywords:** *Pneumocystis jiroveci* infection, Immunocompromised host, Mortality

## Abstract

**Background:**

High-dose steroid therapy has been proven effective in AIDS-related Pneumocystis pneumonia (PCP) but not in non-AIDS-related cases. We evaluated the effects on survival of steroids in HIV-negative patients with PCP.

**Methods:**

Retrospective study patients admitted to the ICU with hypoxemic PCP. We compared patients receiving HDS (≥1 mg/Kg/day prednisone equivalent), low-dose steroids (LDS group, <1 mg/Kg/day prednisone equivalent), and no steroids (NS group). Variables independently associated with ICU mortality were identified.

**Results:**

139 HIV-negative patients with PCP were included. Median age was 48 [40–60] years. The main underlying conditions were hematological malignancies (n=55, 39.6%), cancer (n=11, 7.9%), and solid organ transplantation (n=73, 52.2%). ICU mortality was 26% (36 deaths). The HDS group had 72 (51.8%) patients, the LDS group 35 (25%) patients, and the NS group 32 (23%) patients. Independent predictors of ICU mortality were SAPS II at ICU admission (odds ratio [OR], 1.04/point; [95%CI], 1.01-1.08, P=0.01), non-hematological disease (OR, 4.06; [95%CI], 1.19-13.09, P=0.03), vasopressor use (OR, 20.31; 95%CI, 6.45-63.9, P<0.001), and HDS (OR, 9.33; 95%CI, 1.97-44.3, P=0.02). HDS was not associated with the rate of ICU-acquired infections.

**Conclusions:**

HDS were associated with increased mortality in HIV-negative patients with PCP via a mechanism independent from an increased risk of infection.

## Introduction

*Pneumocystis jiroveci* pneumonia (PCP) is a major cause of acute respiratory failure in immunocompromised patients. Malignant disease, steroid treatment, and transplantation of solid organs or bone marrow are the leading causes of T-cell suppression, which is associated with a high risk of opportunistic infections, including PCP [1]. The number of patients with T-cell suppression has risen in recent years, resulting in an increased incidence of PCP [2,3]. In recent studies, more than 8% of patients with hematological malignancies admitted to the ICU for acute respiratory failure had PCP [4]. Mortality rates of up to 30% have been reported in cancer patients with PCP [5].

Studies done in the 1990s showed that adjunctive treatment with high-dose steroids (HDS) was associated with a dramatic decrease in mortality during PCP episodes in HIV-positive patients [6,7]. Corresponding proof of efficacy is not available for HIV-negative patients with PCP, and findings from the three available studies, all retrospective, are conflicting. In a 1998 study in 30 patients, the 16 patients given adjunctive HDS had no difference in mortality but spent less time on mechanical ventilation compared to the 14 patients managed without steroids [8]. The second study, reported in 1999, compared 15 patients with HDS and 8 without HDS and found no significant differences in mortality or ICU stay length [9]. The most recent study was published in 2011 and found no significant difference in mortality between the 59 patients given HDS and the 29 other patients [9]. More over two retrospective studies from our group could not conclude of outcome improvement with adjunctive steroid in that setting [5-10]. The small sample sizes may have jeopardized the ability of these studies to detect significant differences between patients given HDS and other patients.

The pathophysiology of PCP may differ between patients with and without HIV infection. Studies have shown that HIV-negative patients with PCP were older and had a larger number of co-morbidities [2,11], longer symptom duration at diagnosis [12], and higher neutrophil counts in bronchoalveolar lavage fluid [13], compared to HIV-positive patients [14]. Conceivably, these differences between the two populations might affect the ability of steroid therapy to provide therapeutic benefits.

Here, our objective was to determine whether HDS produced therapeutic benefits in HIV-negative patients with severe PCP requiring admission to the intensive care unit (ICU). To increase the sample size for our investigation, we included patients from four different sources, namely, three previously published studies and a teaching-hospital ICU database.

## Patients and methods

### Patients

We analyzed data from three retrospective studies of HIV-negative patients with *Pneumocystis jiroveci* pneumonia. The first study [9] included HIV-negative patients admitted to two ICUs between 1988 and 1996 for PCP and compared patients who did (n=23) and did not (n=8) receive HDS in addition to standard treatment. The second study described HIV-negative patients with PCP managed in the ICU between 1989 and 1990 and looked for predictors of mortality; 33 of the 39 patients received HDS [10]. Finally, the third study compared 56 cancer patients with PCP to 56 cancer patients with bacterial pneumonia admitted to the ICU between 2001 and 2006; of the patients with PCP, 21 received HDS [5]. Only patients from theses studies who were admitted in ICU were included in the present study. In addition to the data from these three studies, we included data from patients admitted for PCP to the ICU of the Saint Louis Teaching Hospital, Paris, France, between 2006 and 2011.

The IRB from Clermont Ferrand approved data collection for the addition of patients from St Louis hospital in this non interventional study.

Medical chart were reviewed by the investigators (VL or AD) for all included patients . For all four sources of patients, inclusion criteria were age over 18 years, ICU admission, and PCP. Only definite case of PCP were considered (positive IF for pneumocystis or MGG coloration in BAL). Exclusion criteria were patients with HIV infection and patients who were not admitted to the ICU. Moreover, colonized patients defined with positive P. *jiroveci* PCR only, without any pulmonary symptom or treatment of P. *jiroveci* were not included.

### Data collection

The following data were collected for each patient at ICU admission: age, sex, underlying disease, exposure to immunosuppressants, steroid therapy before PCP, prophylactic treatments, time from symptom onset to ICU admission, oxygen requirements, P/F ratio, and underlying disease (as two categories, namely hematological or other malignancy and solid organ transplantation or immunological disease). During the ICU stay, the following were recorded: organ failures, use of mechanical ventilation, use of vasoactive agents, pneumothorax, ICU-acquired infections, and vital status. The P/F ratio was computed in the patients on endotracheal mechanical ventilation and estimated in the other patients. PCP was classified as severe when the P/F ratio at ICU admission was ≤100 and mild when it was >100.

ICU Acquired infection was defined by clinically or microbiologically documented infection during the ICU stay and 48 after ICU discharge. Co infections at admission were defined with another microbiologic agent found in respiratory tests or septicaemia.

On BAL, lymphocytic alveolitis was defined when lymphocyte count was over 10% and polynuclear alveolitis was defined over 5%.

The patients were classified into three groups based on whether they received HDS defined as a steroid dosage ≥1 mg/Kg/day, low-dose steroid therapy (LDS) defined as a steroid dosage <1 mg/Kg/day (HDS), or no steroid therapy (NS) during the ICU stay.

### Statistical analysis

Qualitative variables are described as frequencies (percentage) and quantitative variables as medians (25th-75th percentiles). The primary outcome variable of interest was ICU mortality. Stepwise multivariate analysis was performed to look for patient characteristics at ICU admission that were associated with ICU mortality. For multivariate analysis we used univariate factors with p<0.2 and the three steroid categories forced into the model. The secondary outcome variable of interest was ICU-acquired infection. We compared patients in the three steroid-treatment groups (HDS, LDS, and NS), adjusting for PCP severity at ICU admission.

## Results

**Figure 1:**
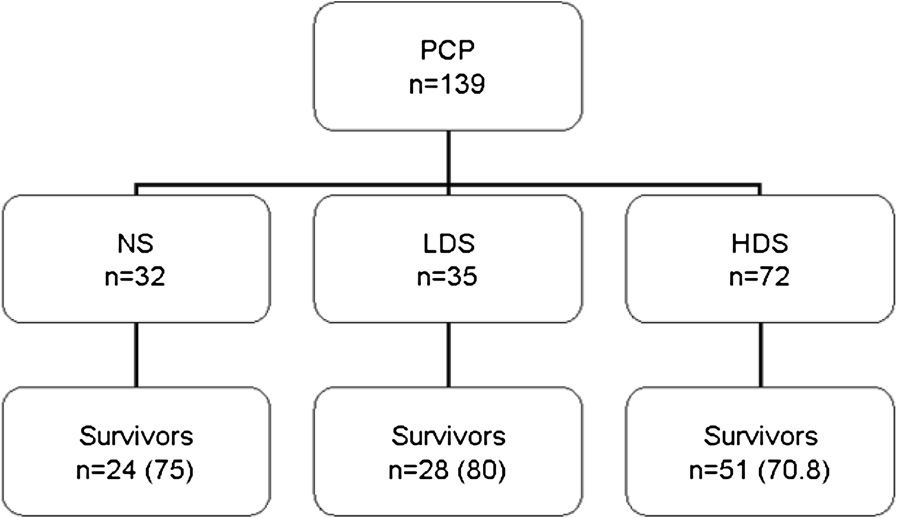
Patients group by steroid use.

From 1988 to 2011, 139 HIV-negative patients were admitted to the ICU with PCP infection (Figure 1). Table 1 described underlying diseases and Table 2 reports main characteristics at ICU admission in survival and dead patients. Co-infection occurred at admission in 28 patients (20%) (Table 3). Mechanical ventilation was required in 84 patients (60%) and vasoactive agents in 41 (29.5%). Time on mechanical ventilation was 0 [0–7] days. Pneumothorax occurred in 11 (7.9%) patients and ICU-acquired infections in 31 (22.3%). Adjuvant steroids were given to 107 (76.9%) patients: 72 (51.8 %) patients received HDS and 35 (25.2%) LDS. The remaining 32 (23%) patients received no steroids in the ICU. More than half of patients received steroids before ICU admission (n=74, 53%), 35 patients in the LDS group and 39 patients in the HDS group. Of the 139 patients, 36 (25.8%) died in the ICU. Length of ICU stay was 8 [4-13] days.

**Table 1:** Underlying disease

**Underlying disease**	**n (%)**
**Hematological malignancy**	84 (60.4)
**Acute myeloid leukemia**	14
**Acute lymphoid leukemia**	7
**Non**-**Hodgkin lymphoma**	25
**Hodgkin lymphoma**	7
**Myeloma**	6
**Chronic myeloid leukemia**	5
**Chronic lymphoid leukemia**	14
**Bone marrow aplasia**	4
**Thrombocytopenic purpura**	2
**Treatment**	
**No chemotherapy**	2
**Chemotherapy only**	52
**Chemotherapy including steroids**	21
**Chemotherapy including fludarabin**	9
**Delay between start of treatment and infection** (**months**)	6 [4-14]
**Solid organ transplantation or immunological disease or cancer**	55 (39.6)
**Solid cancer**	11
**Dermatomyositis**	8
**Solid organ transplantation**	25
**Vasculitis**	2
**Hemolytic anemia**	5
**Other**	4
**Delay between start of immunosuppressive treatment andinfection** (**months**)	6 [4-13]
**Allogenic stem cell transplantation** (**AlloSCT**)	14
**Delay between AlloSCT and pneumocystis infection**(**months**)	7 [4-14]

**Table 2:** Patient characteristics at admission and univariate analysis of risk factors associated with mortality

**Variable**	**Survival patients n=103(75%)**	**Dead patients n=36(25%)**	**p value**
**Age in years,median[IQR]**	46 [39–57]	52 [40–65]	0.15
**Males,n (%)**	61 (59.2)	18 (50)	0.34
**SAPS II,median[IQR]**	31 [23–41]	35 [30–51]	0.01
**Underlying disease, n (%)**			
**Hematological malignancy**	68 (66.0)	16 (44.4)	
**Solid organ transplantation or immunological disease or cancer**	35 (33.9)	20 (55.5)	0.02
**PCP prophylaxis,n (%)**	4 (3.8)	1 (2.7)	0.76
**Steroids before admission, n (%)**	53 (51.4)	21 (58.3)	0.48
**Symptom duration in days, median[IQR]**	7 [3-13]	7 [3-15]	0.71
**Co infection at admission**	20 (19.4)	8 (22.2)	0.66
**P/F ratio at admission**, **median[IQR]**	173 [96-268]	110 [86–197]	0.04
**Mechanical ventilation, n (%)**			
**None**	53 (51.4)	2 (0.5)	
**NIV**	25 (24.2)	1 (0.2)	<0.001
**NIV and MV**	25 (24.2)	33 (91.6)	
**Shock during ICU stay, n (%)**	19 (18.4)	26(72.2)	<0.001
**ICU acquired infection**	14 (13.5)	17 (47.2)	<0.0001
**Steroids in the ICU, n (%)**			
**No Steroid**	33 (32.0)	12 (33.3)	
**Low Dose Steroid**	19 (18.4)	3 (8.3)	0.60
**High Dose Steroids**	51 (49.5)	21 (58.3)	

*IQR* interquartile range, *OR* odds ratio, *95%CI* 95% confidence interval, *SAPS* II, Simplified Acute Physiology Score, version II; *PCPPneumocystis* pneumonia, *NIV* noninvasive ventilation, *MV* endotracheal mechanical ventilation, *ICU* intensive care unit, *HDS* high-dose steroid therapy, *LDS* low-dose steroid therapy, *NS* no steroid therapy.

**Table 3:** Co infections at admission and ICU acquired infection

**Microbiologic documentation**	**Co –infection at admission**	**ICU acquired infections**
	**n= 28(20.1%)**	n=31 (22.3%)*
**Bacterial infections**		
**Pulmonary**	17	7
**Other infections**	2	6
**Mycobacteria**	1	0
**Viral infections**	5	4
**Fungi**		
**Aspergillus**	3	1
**Candidosis**	0	1
**No documented**	na	15

*one patient had more than one infections.

By univariate analysis (Table 2), ICU mortality was associated with age, non-hematological disease, ICU-acquired infection, higher SAPS II, lower P/F, pneumothorax, endotracheal mechanical ventilation, and shock. ICU mortality did not vary significantly across steroid-treatment groups: 29.2% in the HDS group, 20% in the LDS group, and 25% in the HS group (*P*=0.6).

By multivariate analysis, factors independently associated with ICU mortality were steroid use before ICU admission, SAPS II, non-hematological disease, HDS, and shock (Table 4).

**Table 4:** Multivariate analysis of factors independently associated with mortality

**Variable**	**OR [95%CI]**	**P value**
**Steroids before ICU stay**	0.77 [0.17-3.49]	0.74
**Steroids during ICU stay**		
**No Steroid**	5.64 [0.70-45.5]	
**Low Dose Steroid**	1	0.02
**High Dose Steroids**	9.33 [1.97-44.3]	
**SAPS II per point**	1.04 [1.01-1.08]	0.01
**Underlying disease**		
**Hematological disease**	1	
**Other**	4.06 [1.19-13.09]	0.03
**Shock**	20.31 [6.45-63.9]	<0.001

*OR* odds ratio, *95%CI* 95% confidence interval, *ICU* intensive care unit, *HDS* high-dose steroid therapy, *LDS* low-dose steroid therapy* NS* no steroid therapy, *SAPS* II Simplified Acute Physiology Score, version II.

**Figure 2:**
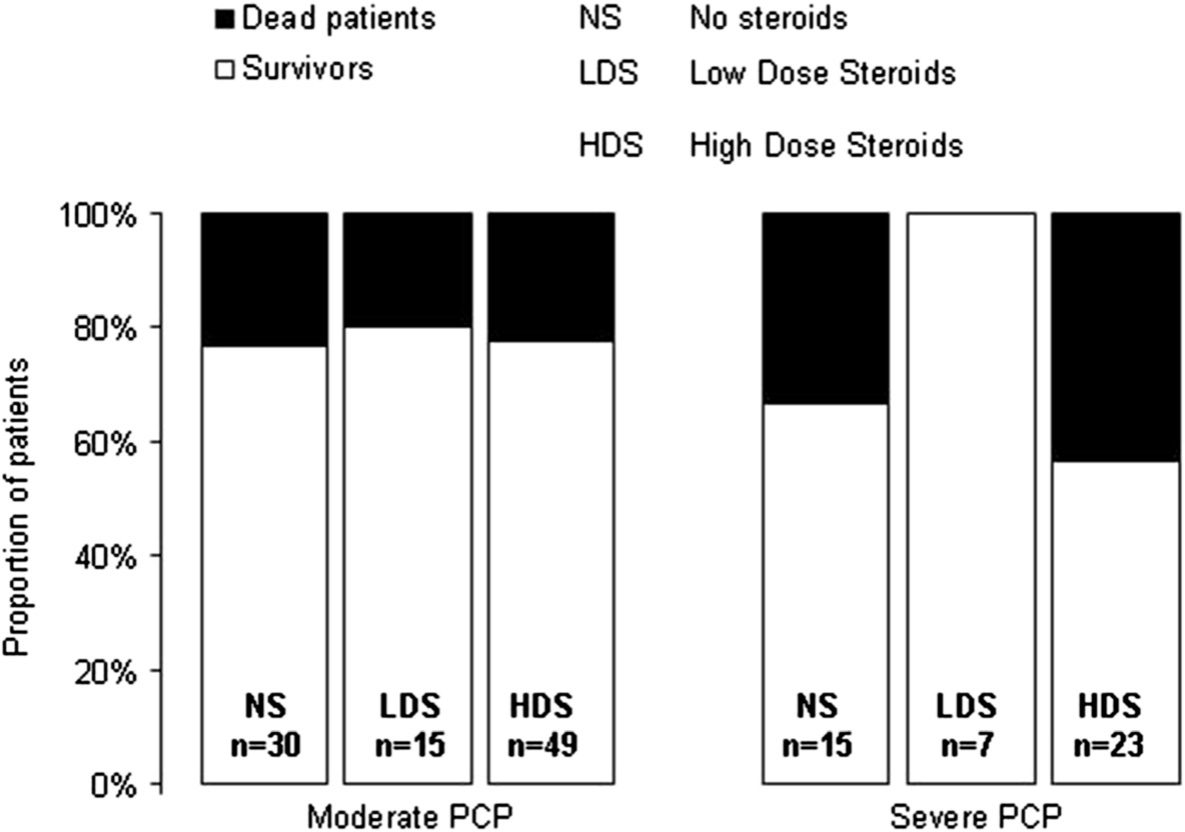
Outcome according severity of pneumocystic pneumonia and adjunction steriods therapy.

In the group of 59 patients who required endotracheal mechanical ventilation, ICU mortality was not significantly different between the HDS patients (20/30 patients died, 66%) and the other patients (13/29 died, 45%) (*P*=0.09). Figure 2 described outcome according the severity of PCP and steroid group.

ICU acquired infection occurred in 31 (22.3%) patients (Table 4), respectively in 12.5%, 31.4% and 22.2% in no steroid group, LDS group and HDS group, respectively (p=0.19).

By multivariate analysis, independent risk factors for ICU-acquired infection were shock (OR= 6.80; [2.55-18.14], *p*<0.001) and pneumothorax (OR, 4.80; [1.09-21.1], *p*=0.04). The risk of ICU-acquired infection was not influenced by the SAPS II (*p*=0.36), steroid treatment before ICU admission (*p*=0.91), or steroid treatment in the ICU (*p*=0.46).

A comparison of three time periods (1991–1996, 1997–2004, and 2005–2011) showed a trend for declined in mortality over time (respectively 36%, 26%, 18%; p=0.12). The proportion of patients managed with endotracheal mechanical ventilation was higher in the most recent period (respectively 36%, 40%, 44%; p=0.73), whereas the proportion of patients given HDS was higher in the earliest period (respectively 83%, 48%, 33%; p<0.001).

Data on BAL count were available for 47 patients: 13 patients had neutrophil alveolitis (>10%), 7 patients had lymphocyts alveolitis (>5%), 4 patients had normal BAL count and 21 had alveolitis with neutrophils and lymphocyts.

## Discussion

In this study, HDS was associated with an increase in ICU mortality in HIV-negative patients with PCP. This increase was not related to a higher rate of ICU-acquired infection.

In recent non-ICU studies of patients with PCP, up to 30% of patients died [15]. However, the 25.8% overall ICU mortality rate in our study compares favorably with the 39% to 66% rates in other ICU studies done between 1995 and 2011, with the highest rates being reported in mechanically ventilated patients [2,11,15]. During the same period, survival improved overall among immunocompromised patients admitted to the ICU [16,17].

The pathophysiology of PCP may differ between HIV-positive and HIV-negative patients. For instance, differences in lymphocyte and neutrophil counts in bronchoalveolar lavage fluid have been reported [13,14]. In our study, in agreement with Limper et al. most BAL included neutrophil alveolitis. [13]. Diagnosis remained easier in HIV infected patients with higher organism burden. [15]. In ICU studies, PCP was more often fatal in non-HIV-infected patients than in HIV-positive patients [2,11]. The HIV-positive patients were younger and had less co-morbidity [11]. Similarly, our patients were older than those in studies of HIV-positive patients. We did not collect co-morbidities. Co-infections, most notably with viruses, were considerably more prevalent among HIV-negative patients than HIV-positive patients in previous studies [11,18].

In HIV-negative patients with PCP, there is no proof that adjunctive steroid therapy is beneficial [5,8-10]. The most recent study compared 29 patients treated with adjunctive steroid therapy to 59 patients treated without steroids [16]. Adjunctive steroid therapy was defined as at least 40 mg of prednisone *bid*, and no effort was made to separate patients on HDS and LDS. The proportions of patients with hematological malignancies and solid organ transplants were similar to those in our population. ICU mortality was 35.8% overall, with no significant difference between patients managed with and without steroids. In our study, mortality was lower (25.8%), despite greater acute illness severity at ICU admission for acute respiratory failure. Adjunctive HDS was independently associated with increased mortality in our study. Steroid therapy might be expected to increase the rate of viral reactivation and bacterial infection in immunocompromised patients [18]. However, the adverse effect of HDS on mortality was not related to an increase in the rate of ICU-acquired infections. ICU-acquired infections occurred in only 22% of our patients, a low rate given their immunocompromised status.

Our study has several limitations. First, only 139 patients were included over a 23-year period. Outcome of severe PCP tends to improve throughout this study period and more patients in the earlier period received HDS. PCP in immunocompromised patients has become uncommon since the use of routine prophylactic treatment [19,20]. In addition, we confined our study to patients with severe illness requiring ICU admission. Second, we used a retrospective design. We had no information on the factors that governed the use and timing of steroid therapy. Moreover, steroid therapy seemed less beneficial in the patients with greater disease severity (Figure 2). We cannot exclude that HDS was started in some patients as rescue therapy after endotracheal mechanical ventilation became necessary. Nevertheless, our study is the largest in this field [8,16] and showed that HDS was independently associated with mortality. Third, we did not have complete data collected on the day of steroid therapy initiation. Fourth, some patients were included many years ago, at a time when the proportion of patients given HDS was higher than in recent years. In this early period, few tests were available for detecting viral infections. Viral co-infection may increase ICU mortality and the risk of ICU-acquired infections [2,18]. Moreover, ICU mortality of immunocompromised patient improved over time [17,21]. Fifth, we separate patients in three groups according the steroids dose, resulting in a limited number of patients in each group. Particularly, in LDS group mortality was the lowest but not significantly different from other group due to the small number of patients.

## Conclusions

In this study, adjunctive HDS in HIV-negative patients admitted to the ICU with mild-to-severe PCP was associated with an increased risk of ICU mortality. This increase in mortality was not related to a higher rate of ICU-acquired infection in this retrospective study. A prospective is warranted to confirm these results.

## Abbreviations

AIDS: Acquired immunodeficiency syndrome; HDS: High-dose steroids; HIV: Human immunodeficiency virus; ICU: Intensive care unit; LDS: Low-dose steroids; NS: No steroids; PCP: *Pneumocystis* pneumonia; P/F: Ratio of arterial oxygen tension over fraction of inspired oxygen.

## Competing interests

The authors declare that they have no competing interests.

## Author’s contributions

VL, AD, EA, designed the study, collected and interpreted the patient data and wrote the manuscript, AD, CA, analyzed the data. All authors read and approved the final manuscript.
